# Developing Improved Corrosion-Resistant AA5083—BN/WC Composites for Tribological Applications

**DOI:** 10.3390/ma16041663

**Published:** 2023-02-16

**Authors:** Hany R. Ammar, Elsayed M. Sherif, Subbarayan Sivasankaran, Fahad A. Almufadi, Abdel-baset H. Mekky

**Affiliations:** 1 Department of Mechanical Engineering, College of Engineering, Qassim University, Buraydah 51452, Saudi Arabia; 2 Center of Excellence for Research in Engineering Materials (CEREM), Deanship of Scientific Research, King Saud University, Riyadh 11421, Saudi Arabia; 3 Department of Physics, College of Science and Arts El-Meznab, Qassim University, Buraydah 51931, Saudi Arabia

**Keywords:** AA5083-matrix composite, tungsten carbide (WC), boron nitride (BN), corrosion passivation, chloride solutions, electrochemical methods

## Abstract

In this study, corrosion-resistant AA5083—BN/WC composites were developed for tribological applications through adequate control of the reinforcement content (WC and BN) in the matrix (AA5083 alloy). The effects of 6% and 12% tungsten carbide (WC) as well as 6% and 12% boron nitride (BN) additions on the corrosion behavior of AA5083 aluminum composite in 3.5% NaCl solution were carried out. Electrochemical techniques such as cyclic potentiodynamic polarization (CPP), changes in the chronoamperometric current with time (CCT), and electrochemical impedance spectroscopy (EIS) were utilized. The polarization results showed that the addition of 6% WC to the AA5083 alloy matrix improved its resistance to corrosion (R_P_). Rp exhibited an additional increase by adding 12% WC to the matrix. The values of R_P_ were observed to increase for the AA5083 composite when adding 6% BN, and the highest R_P_ values were recorded for the composite that contains 12% BN. The results obtained by the CPP method were confirmed by CCT and EIS measurements, where the presence of WC and BN protected the developed AA5083- BN/WC composites against corrosion. The corrosion resistance revealed an additional improvement with an increase in WC and BN content from 6% to 12%. The results also confirm that pitting corrosion decreased in the presence of WC and BN in the fabricated composites.

## 1. Introduction

Aluminum and its alloys have been successfully employed in electronic technology, electric module packaging, wind and solar energy management, alkaline batteries, and automotive body structures, including body parts, radiators, engine castings and wheels. All these applications are related to the characteristics of Al alloys, such as their high specific strength, high toughness, ease of welding, good conductivity, high corrosion resistance in the atmosphere, anti-erosion, high processability, eco-friendly nature, and recoverability [1,2,3]. However, the tribological properties of the AA5083 alloy need improvements to meet several application requirements, such as a low friction coefficient and high wear resistance, because these alloys are subject to dynamic contact loads and the relative motion of contact surfaces. The improved tribological behavior of the AA5083 alloy requires enhancing the corrosion resistance to ensure high-quality surface properties that improve the tribological behavior of such materials.

Al alloy 5083 (AA5083) has been employed in marine environments because of its considerable corrosion resistance [4]. This application of the alloy is threatened by pitting corrosion, which occurs due to the metal’s reaction with chloride ions in the marine environment. Several studies [4,5,6,7,8] have proposed that the formation of a protective layer on the AA5083 surface, as in the case of other aluminum alloys, provides better corrosion resistance by preventing its dissolution in the corrosive electrolyte.

AA5083 metal matrix composites (AA5083-MMCs) were developed to improve the corrosion resistance and mechanical properties of AA5083 to meet several application requirements. Kaoru et al. [9] reported that alloying AA5083 with Mg strengthened it because of the solid solution strengthening mechanism. The same principle was confirmed by Nam et al. [10], who claimed that the presence of Mg facilitated the formation of Al_2_O_3_ and MgO and thus increased the passivation of the alloy. The incorporation of the Al-N composite and Mg within the alloy reduces the size of the grains in the alloy matrix, which improves its pitting corrosion [11]. The role of adding several metal oxides to enhance the corrosion behavior of the AA5083 alloy has been reported [12,13,14,15,16,17]. The addition of boron carbide, B_4_C, to alloy 5083 to form Al-B_4_C metal matrix composites has been reported to enhance the mechanical performance and corrosion behavior of AA5083 alloys [12]. The use of other metal oxides, such as Al_2_O_3_ [13], TiO_2_ [14], SiC [15], TiC [16], and ZrO_2_ [17], revealed similar effects. In these studies, the addition of these particles was beneficial for enhancing the corrosion and wear resistance and improving the mechanical characteristics.

Reinforcing aluminum with submicron TiC and WC and their effects on the wear behavior were reported [18]; the presence of WC was observed to improve the wear behavior of aluminum. The influence of 1, 2 and 3 wt.% WC on the microhardness, roughness, and corrosion of Al-12Si alloy in 0.2M HCl solution was investigated by H. A. Abdullah [19]. It was found that the corrosion resistance was remarkably improved by WC compared to the base alloy [19]. The Al-BN system was also tested in a chloride solution to evaluate the galvanic corrosion performance [20].

Boron nitride (h-BN type), with a lamellar structure similar to graphite, exhibits several interesting physical and mechanical properties. It exhibits high thermal conductivity, excellent chemical stability, superior solid lubrication behavior, reduced wear and friction characteristics, and self-lubricating behavior. These properties make h-BN an interesting addition for improving tribological properties [21,22,23,24,25,26].

This study aimed to develop highly corrosion-resistant AA5083– BN/WC composites for tribological applications. It investigated the effect of adding 6 wt.% and 12 wt.% of tungsten carbide (WC) and boron nitride (BN) on the corrosion of the AA5083 alloy matrix in 3.5% sodium chloride solutions. The investigation was performed using advanced electrochemical and spectroscopic methods. Cyclic potentiodynamic polarization was used to measure the corrosion potentials and corrosion currents from which the corrosion rates and corrosion resistance were calculated. Kinetic parameters related to the transfer of electrons at the interface of the composite surface were reported using electrochemical impedance spectroscopy. Particular attention was given to the examination of the surface for pitting corrosion by applying two different active anodic potential values and evaluating the output currents with time.

## 2. Materials and Methods

### 2.1. AA5083-WC and AA5083-BN Composites

The composites were fabricated through a powder metallurgy route in which starting powders of aluminum alloy (AA5083), tungsten carbide (WC), and boron nitride (BN) were used for ball milling (BM) to fabricate AA5083-WC and AA5083-BN composites with different levels of WC and BN reinforcements. AA5083 powders exhibited an average size of −325 mesh, the hexagonal BN powders revealed an average size of −325 mesh and 99.50% purity, and WC powders displayed an average size of −100 + 270 mesh and 99.00% purity.

AA5083-WC and AA5083-BN composites were synthesized using the Pulverisette 5/2 classic line ball milling technique. The starting powders of the AA5083 alloy, WC, and BN were prepared in a premixed form according to the designed composition. The powders of each composite were charged in two tungsten carbide containers with 250 mL capacity each. Tungsten carbide balls with diameters of 10 mm were used as grinding media. The composite powders were processed using ball milling (BM) by applying the following milling regime: 5 h milling time, 250 rpm milling speed, and 10:1 ball-to-powder mass ratio. The dry milling process was performed under an inert atmosphere of argon gas to protect the processed powders from oxidation. Then, 2 wt.% of stearic acid was added to the mixed powders to avoid cold welding. For each composite, the BM process was conducted for 5 h according to a repeated cycle, including 15 min milling, 15 min of stopping, 15 min milling in the reverse direction, and 15 min of stopping. Each BM cycle comprised two breaks for 15 min/each to reduce heat accumulation inside the containers. Moreover, the BM cycles included forward/reverse milling to ascertain the homogeneous distribution of WC and BN powders in the AA5083 matrix.

The developed composites in the powder form were hot-consolidated using the forging-sintering technique, where the composites were heated to 550 °C for 30 min and immediately hot-forged at 225 MPa. The load was kept for three minutes as a holding time. All bulk composites displayed a relative density of more than 97%. Table 1 shows the reference and actual compositions of the as-received AA5083 alloy used in the present study, while Table 2 shows the designed composition of the fabricated composites in terms of weight percentage. In addition, sodium chloride salt was purchased from Sigma Aldrich (99.99% purity) and used to prepare a 3.5% NaCl solution for corrosion experiments in the present study.

### 2.2. Electrochemical Techniques

For all electrochemical experiments, an electrochemical cell containing 100 mL of NaCl solution was used. This cell also accommodates the rods of the tested alloys. A platinum (Pt) sheet and silver/silver chloride (Ag/AgCl) were used as the working, counter, and reference electrodes, respectively. The working electrodes were cylindrical with an area of 1 cm^2^. For electrochemical measurements, the alloy samples were ground, welded, and mounted in epoxy, as reported in previous studies [27]. Metrohm Autolab Ecochemie PGSTAT-302N was used. CPP plots were collected between −1400 and −500 mV at a scan rate of 0.00167 mV. The potential was scanned from the more negative values to the less negative value in the backward direction until the current obtained intersected with the one in the forward direction from −1500 mV to −500 mV and rescanned until the backward potential intersected with the forward one. The CCT current-time plots were acquired at two fixed values of potentials, one at −750 mV and another at −680 mV, which were applied to the different composites for 2250 s. The EIS spectra were gathered from the value of corrosion potential in a frequency range of 100,000–0.10 Hz [1]. The CPP, CCT, and EIS data were measured at room temperature after 60 min immersion in the 3.5% NaCl test electrolyte.

## 3. Results

### 3.1. Cyclic Potentiodynamic Polarization (CPP) Results

**Figure 1:**
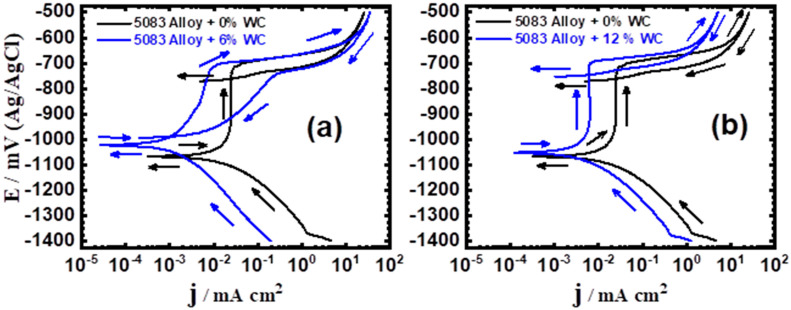
CPP curves for AA5083-WC composites immersed in 3.5% NaCl solution for 60 min: (**a**) AA5083 versus AA5083 + 6% WC; and (**b**) AA5083 versus AA5083 + 12% WC.

**Figure 2:**
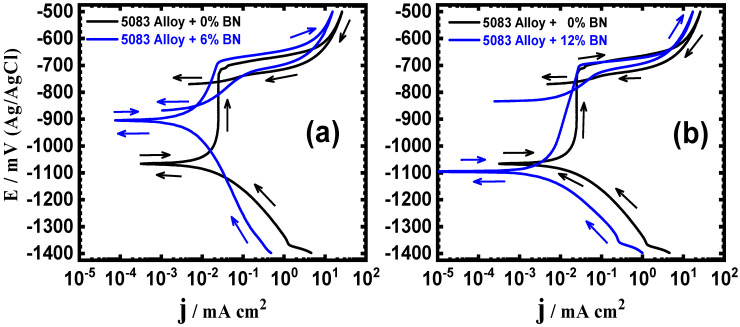
CPP curves for AA5083-BN composites immersed in 3.5% NaCl solution for 60 min: (**a**) AA5083 versus AA5083 + 6% BN; and (**b**) AA5083 versus AA5083 + 12% BN.

The CPP experiments were utilized to investigate the influence of WC and BN content on the corrosion of the AA5083-matrix composites in a 3.5% NaCl solution. The polarization technique has long been widely utilized to examine the corrosion of materials in corrosive media [27]. Figure 1 displays the curves of CPP for the AA5083-WC composites: (a) 0% WC vs. 6% WC and (b) 0% vs. 12% WC. Figure 2 shows the same measurements carried out for the AA5083-BN composites: (a) 0% BN vs. 6% BN and (b) 0% BN vs. 12% BN. The polarization parameters extracted from Figure 1 and Figure 2 are listed in Table 3. The data in the table include the cathodic Tafel slope (βc), corrosion potential (E_Corr_), anodic Tafel slope (βa), corrosion current (j_Corr_), protection potential (E_Prot_), pitting potential (E_Pit_), polarization resistance (R_P_), and corrosion rate (R_Corr_). The values of βc, βa, E_Corr_, and j_Corr_ were obtained as previously described in the preceding studies [27]. E_Prot_ is the potential after which pitting corrosion starts to occur, and it is the potential at which the reversed anodic branch intersects with the forward anodic branch [28]. In addition, the E_Pit_ is the potential at which the surface protective layer starts to dissolve and break down, causing a rapid increase in the current values in the anodic branch and resulting in the appearance of pitting corrosion [28]. Furthermore, R_P_ and R_Corr_ values were calculated using Equations (1) and (2), respectively [27];

(1) $$R_{P}=\frac{1}{j_{\mathrm{Corr}}}\;\left(\frac{\beta_{c}.\beta_{a}}{2.3\left(\beta_{c}+\beta_{a}\right)}\right)$$

(2) $$R_{\mathrm{Corr}}={\;j}_{\mathrm{Corr}}\left(\frac{k.E_{W}}{d.A}\right)$$

The definition of these parameters are as follows: k is a constant = 3272 mm (amp^−1^ cm^−1^ year^−1^)); E_W_ refers to the equivalent weight of the AA5083 alloy, which equals 26.50 g equivalent; d indicates the AA5083 alloy density, which equals 2.65 gm cm^−3^; and A is the surface area of the material, which equals 1 cm^2^.

For all the composites, the CPP curves show that the current diminishes in the cathodic branch until the current reaches the value of j_Corr_. This can be attributed to the reduction of oxygen on the composite surface because it receives a less negative potential and is immersed in a near-neutral solution containing oxygen. This reaction may proceed in this way [28,29]:

2H_2_O + O_2_ + 4e^−^ = 4OH^−^ (3)

It has been reported that the formation of OH- can follow this reaction [30]:

(4) $$\frac{1}{2}O+H_{2}O+2^{-}=2{\mathrm{OH}}^{-}$$

The strength of the cathodic reaction under this condition depends on the percentage of reinforcement, WC, and BN, as well as the corrosiveness of the chloride test solution. However, the anodic currents are obtained while applying a less negative potential, and this increases after obtaining the value of j_Corr_. The current increase is related to surface layer dissolution, which may proceed as follows [6];

Al = Al^3+^ + 3eˉ (5)

After the dissolution of Al, the current did not show a significant increase, and a passive region appeared because of the adsorption of the hydroxide ions in Equation (3), which resulted from the cathodic reaction that occurred in the AA5083 alloy matrix. The reaction proceeds as follows [2,3]:

(6) $${\mathrm{Al}}_{\left(S\right)}+3{\mathrm{OH}}^{-}=\mathrm{Al}{\left(\mathrm{OH}\right)}_{3,\mathrm{ads}}+3e^{-}$$

The formed (Al(OH)_3_) was rapidly converted into hydrated aluminum oxide $\left({\mathrm{Al}}_{2}O_{3}.3H_{2}O\right)$ as per the following reaction [2,3,30]:

(7) $$2\mathrm{Al}{\left(\mathrm{OH}\right)}_{3,\mathrm{ads}}={\mathrm{Al}}_{2}O_{3}\cdot 3H_{2}O$$

The continuous application of a less negative potential (anodic branch) helps build the oxide (Equation (8)) until a potential value (E_Pit_); then, the current increases, causing the breakdown of this oxide, and pitting corrosion may occur. The incidence of pitting corrosion is confirmed by the presence of a hysteresis loop in the CPP curves. This loop appears as a result of the higher current values obtained when the potential was applied in the opposite direction (from less negative towards more negative) in the anodic branch. The increase in the hysteresis loop size indicates a stronger severity of pitting corrosion. This pitting attack occurs because the chloride ions in the solution attack the defective areas of the formed oxide, reaching the surface of the AA5083 composites and forming AlCl_3_, according to Equation (8):

Al^3+^ + 3Cl^−^ = AlCl_3_ (8)

The continuous attack on the surface by Cl^−^ leads to the formation of an aluminum chloride complex, AlCl_4_^−^ [29,30,31]. This complex diffuses, leaving the surface into the bulk solution [30,31,32,33]:

Al + 4Cl^−^ = AlCl_4_^−^ + 3e^−^ (9)

From Figure 1a, it may be seen that the addition of 6% WC decreased both the cathodic and anodic currents. The data displayed in Table 3 confirmed these observations, where the addition of 6% WC decreased the values of j_Corr_ and R_Corr_ and increased the resistance, R_P_. The addition of 12% WC, Figure 1b, increased the corrosion resistance by decreasing all corrosion parameters, as seen in Figure 1 and listed in Table 3. The same effect was obtained when adding 6% BN and 12% BN (Figure 2a,b, respectively), where these contents increased the corrosion resistance of the AA5083-BN composites by decreasing their corrosion rate. Comparing the polarization data collected for the tested composites in the presence of WC to the results obtained for the composites in the presence of BN, it was found that BN has a slightly better effect on enhancing corrosion resistance and reducing the current and rate of corrosion. In addition, the presence of WC increased the resistance to pitting corrosion, and this behavior was even increased in the presence of BN. This occurs by shifting the pitting potential to a lesser negative value, as can be seen from the values of E_Pit_ in Table 3. The attained results confirmed that the presence of 6% WC and 6% BN improved the corrosion resistance. A further increase in the reinforcement addition (12% WC and 12% BN) led to an additional increase in the corrosion resistance of the developed composites.

### 3.2. Change of Current with Time Results

**Figure 3:**
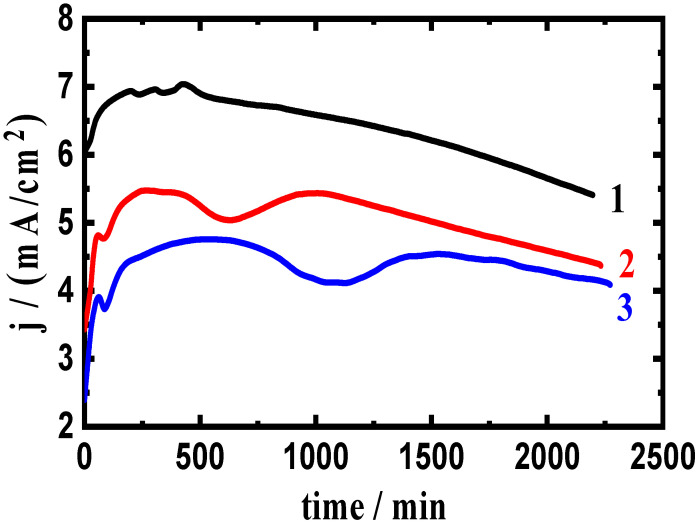
Current curves collected at −750 mV for AA5083 composites with (1) 0% WC, (2) 6% WC, and (3) 12% WC immersed in 3.5% NaCl solution for 60 min.

**Figure 4:**
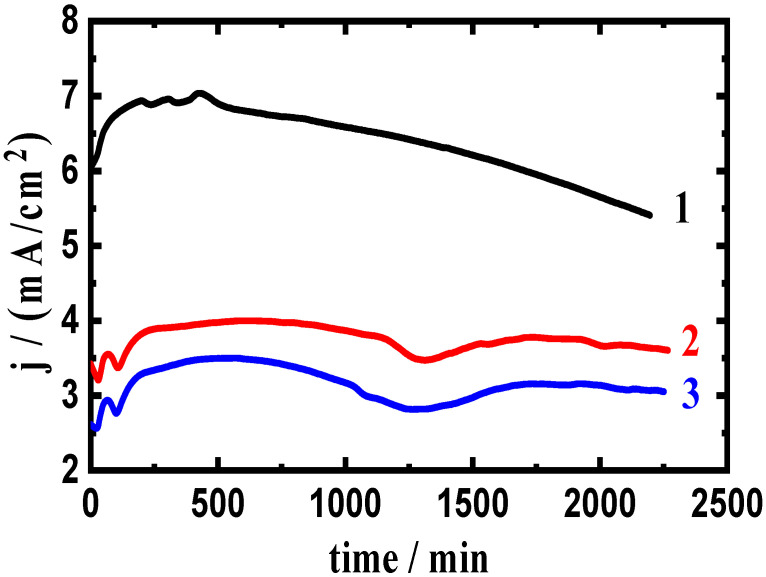
Current curves collected at −750 mV for AA5083 composites with (1) 0% BN, (2) 6% BN, and (3) 12% BN immersed in 3.5% NaCl solutions for 60 min.

The variation in the chronoamperometric current with time (CCT) experiments at two anodic potential values, −750 mV and −680 mV (Ag/AgCl), were utilized to express the corrosion of the tested composites. This technique has been successfully used to investigate corrosion, particularly the pitting type in various electrolytes [2,3,34,35]. The CCT experiments were carried out at −750 mV and −680 mV, which were chosen from the anodic branch of the polarization curves presented in Figure 1 and Figure 2 in the region of pitting corrosion. The CCT curves for AA5083 composites with (1) 0% WC, (2) 6% WC, and (3) 12% WC at −750 mV are shown in Figure 3. Figure 4 shows the results obtained for the composites with (1) 0% BN, (2) 6% BN, and (3) 12% BN. The measured current for the AA5083 alloy matrix without WC and BN (curve 1) was almost 6 mA/cm^2^ at the first moment of the alloy immersion. This value increased over the first 500 s, indicating that the surface of the alloy dissolved, as shown in Equation (5). Moreover, according to the opinion of Foley et al. [36] and Diamanti et al. [37], Cl^−^ ions are chemisorbed onto the surface oxide layer of the materials immersed in the chloride solution. This process dissolves the oxide layer, resulting in the formation of an oxychloride complex as follows:

Al + 4Cl^−^ = AlCl_4_^−^ + 3e^−^ (10)

Thereafter, the current slightly decreases until the end of the run because of the formation of a corrosion product on the material surface. The AA5083-6%WC composite (Figure 3, curve 2) showed a similar behavior but with significantly decreased overall current values, and this effect was further increased when increasing the composite content to 12% WC (Figure 3, curve 3). The addition of WC (AA5083-6%WC and AA5083-12%WC composites) resulted in decreasing the absolute current and decreased the corrosion of the AA5083-WC composite. A greater reduction in corrosion was observed when the WC content increased from 6% to 12%. A greater decrease in the current values was obtained for the AA5083 composite when adding 6% BN and 12% BN. Comparing the effect of adding WC and BN on the current values of the AA5083 composites, the presence of BN revealed a higher corrosion resistance. However, both WC and BN prevented pitting corrosion and decreased the severity of general corrosion when −750 mV was applied as a fixed potential value for 2300 s.

**Figure 5:**
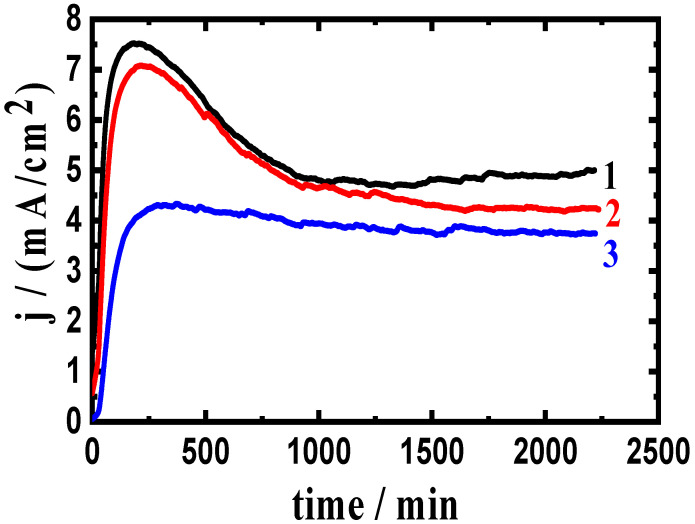
Current curves collected at −680 mV for AA5083-WC composites: (1) 0% WC, (2) 6% WC, and (3) 12% WC immersed in 3.5% NaCl solution for 60 min.

**Figure 6:**
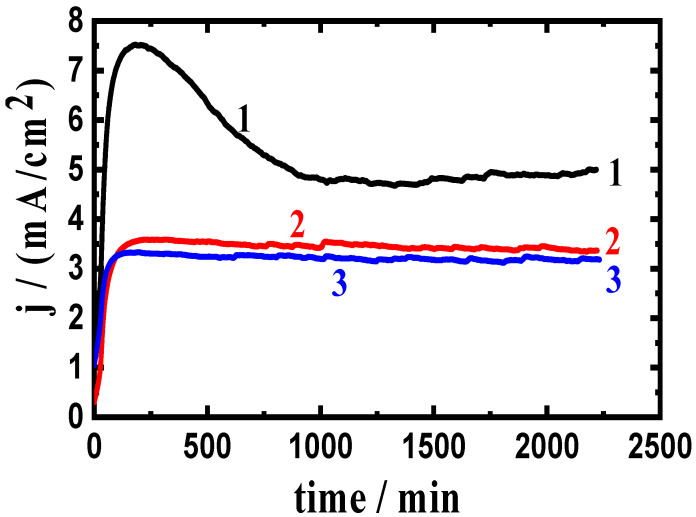
Current curves collected at −680 mV (Ag/AgCl) for AA5083-BN composites: (1) 0% BN, (2) 6% BN, and (3) 12% BN immersed in 3.5% NaCl solution for 60 min.

A potential of −680 mV was applied for 2300 s to examine the influence of changing the anodic potential to a less negative value on the measured current versus time. Figure 5 shows the obtained results at −680 mV for (1) AA5083-0%WC, (2) AA5083-6% WC, and (3) AA5083-12% WC composites. Figure 6 shows similar curves obtained at the same potential and time for (1) AA5083-0% BN, (2) AA5083-6% BN, and (3) AA5083-12% BN composites. Figure 5 and Figure 6 display that the recorded initial currents were almost zero. The current then rapidly increased to the highest values within approximately 200 s, which was mostly due to the dissolution of the created oxide layer during the immersion of the material for 60 min before using −680 mV as the anodic potential. The current decreased slightly thereafter and for almost 1000 s because of the creation of a corrosion layer on the composite surface. The current finally stabilized until the end of the experiment owing to the thickening of the created layer of the corrosion product. It is also seen that the alloy matrix without any WC or BN content (Curve 1 in Figure 5 and Figure 6) recorded the highest current values. The presence of 6% WC (curve 2 in Figure 5) slightly reduced the collected currents, indicating that 6% WC provided a little protection against the corrosion of the AA5083-WC composite. This effect becomes stronger when a 12% WC is added to the matrix (curve 3, Figure 5), where the current remarkably decreased, confirming that the presence of 12% WC provided great protection against corrosion for the AA5083-WC composite in NaCl solutions.

The effect of adding BN is shown in Figure 6, where the addition of 6% BN (curve 2) greatly decreased the absolute current from almost 7.5 mA/cm^2^ to circa 3.5 mA/cm^2^ at the beginning. A similar effect was also collected for the composite with 12% BN but with slightly lower values of the recorded currents. This indicates that the presence of BN in the investigated contents remarkably reduced the uniform corrosion of the AA5083-BN composite, as indicated by the lower absolute current values. Compared to the current-time behavior obtained for the AA5083-WC composites, the absolute currents recorded in the presence of BN are much lower. The results of the CCT at −680 mV confirmed the data obtained at −750 mV and also agreed with the results of the CPP measurements, the results of which showed that the presence of WC reduced the corrosion of the AA5083-matrix composite, and the presence of BN provided slightly better passivation.

### 3.3. Electrochemical Impedance Spectroscopy (EIS) Results

**Figure 7:**
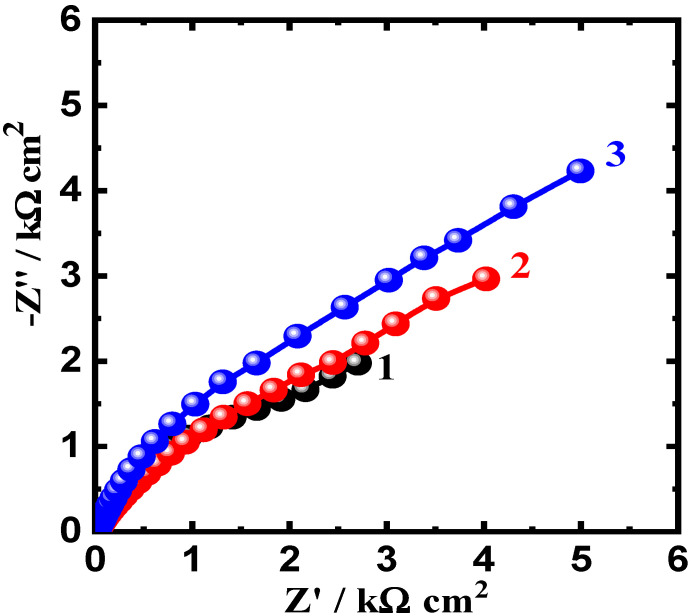
Nyquist plots for AA5083-matrix composite with (1) 0% WC, (2) 6% WC, and (3) 12% WC immersed in 3.5% NaCl solution for 60 min.

**Figure 8:**
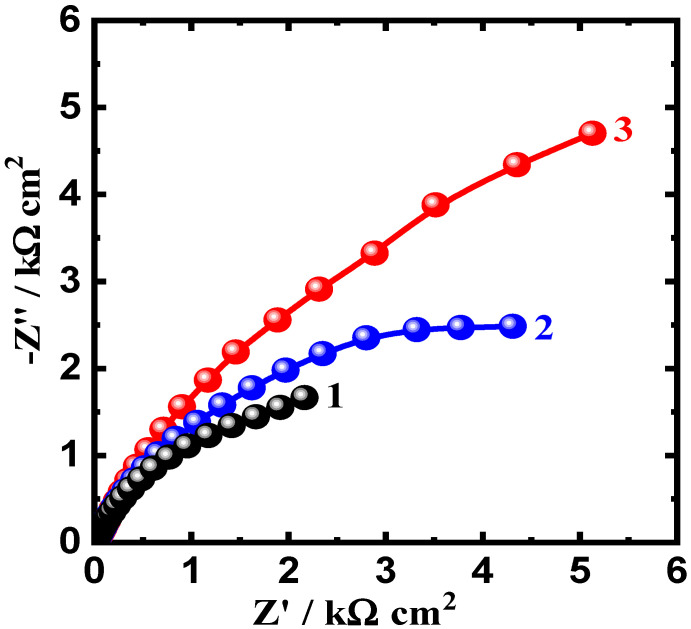
Nyquist plots for AA5083-matrix composite with (1) 0% BN, (2) 6% BN, and (3) 12% BN immersed in 3.5% NaCl solution for 60 min.

**Figure 9:**
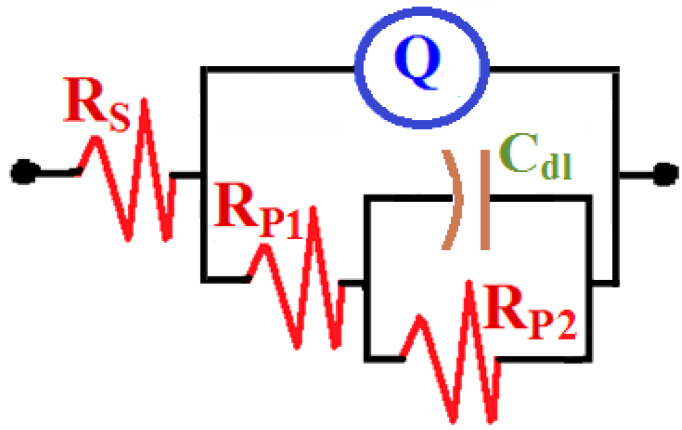
The corresponding circuit used to fit the impedance data.

EIS tests were carried out to investigate the influence of WC and BN levels on the corrosion resistance of AA5083-WC/BN composites in chloride solution and to confirm the results obtained by CPP and CCT measurements. The Nyquist plots of AA5083-WC composite with (1) 0% WC, (2) 6% WC, and (3) 12% WC in 3.5% NaCl solution are displayed in Figure 7. The Nyquist plots obtained for the AA5083-BN composites with (1) 0% BN, (2) 6% BN, and (3) 12% BN are shown in Figure 8. The data obtained from Figure 7 and Figure 8 were fitted to the corresponding circuit displayed in Figure 9 and were employed to fit the impedance data for similar systems [27,35,38,39]. The values of the circuit components are listed in Table 4. This equivalent circuit comprises the resistance of the solution (R_S_), constant phase elements (CPEs) (Q), first polarization resistance (R_P1_), double layer capacitance (C_dl_), and second polarization resistance (R_P2_).

From Figure 7 and Figure 8, only semicircles can be observed. The diameter of this semicircle was the smallest for the AA5083-matrix without addition (0% WC or 0% BN). Adding 6% WC (Figure 7, plot 2) increased the diameter, which was significantly increased by the addition of 12% WC. The data listed in Table 4 reveal that the surface and polarization resistances, R_S_, R_P1_, and R_P2_, recorded the lowest values for the alloy matrix. On the other hand, 6% WC addition increased these values, and further increases were observed for the composite with 12% WC. The values of the Y_Q_ diminished with the presence of WC and with an increase in its content. Furthermore, the value of the exponent n that accompanies Q is close to 0.5, which means that Q may represent a Warburg impedance, and its presence indicates the passivation of the composite surface because of the presence of WC within the alloy matrix. As previously reported [40,41,42], the Q CPEs herein can be defined as follows:

(11) $$Z_{CPE}=\frac{Y0-1}{\left(j\omega \right)-n}$$

Y_CPE_ = −Y_0_(jω)^n^ (12)

Z_CPE_ = [Q(2πfi)^n^]^−1^ (13)

The elements in Equations (11)–(13) can be defined as follows: Y_0_ is the CPE constant; ω is the angular frequency (rad S^−1^); and j^2^ = −1 is an imaginary number. The values of C_dl_ also decreased in the presence of WC, which gives another proof of the protection of the surface of the composite, and this protection was further improved by increasing WC content from 6% to 12%. Figure 8 displays the Nyquist spectra for AA5083-BN composites, where the addition of 6% BN increased the diameter of the obtained plot, and a larger diameter for a semicircle (Plot 3) was obtained for the composite with 12% BN. This behavior confirms that the presence of BN at 6% passivates the surface, whereas the highest protection under this condition is achieved in the presence of 12% BN.

**Figure 10:**
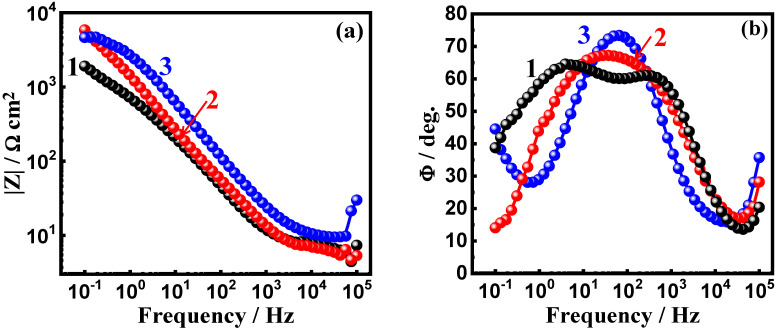
Bode (**a**) impedance and (**b**) phase angle degree plots for AA5083-matrix with (1) 0% WC, (2) 6% WC, and (3) 12% WC immersed in 3.5% NaCl solution for 60 min.

**Figure 11:**
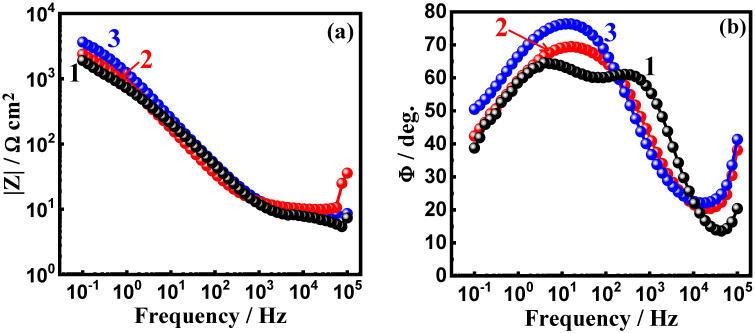
Bode (**a**) impedance and (**b**) phase angle degree plots for AA5083-matrix with (1) 0% BN, (2) 6% BN, and (3) 12% BN immersed in 3.5% NaCl solution for 60 min.

The impedance measurements were also plotted in the form of the Bode impedance of the interface |Z| and the phase angle degree (Φ) to confirm the effects of the addition of WC and BN on the corrosion passivation of the AA5083-WC/BN composite in the test solution. The plots in Figure 10 show the Bode (a) |Z| and (b) Φ for the AA5083-matrix composite with (1) 0%WC, (2) 6% WC, and (3) 12% WC. The same measurements were performed for the AA5083-BN composites, and the Bode plots are shown in Figure 11. The value of |Z| in Figure 10a was augmented with the decrease in the frequency with or without WC. Here, the matrix without WC had the lowest |Z|, whereas the presence of 6% WC shifted the absolute values for |Z|, but the highest values are noticed for the AA5083 composite with 12% WC. Figure 10b, in turn, shows that the maximum values of Φ are observed for the composite with 12% WC. The Bode plots in Figure 11 reveal the same effect for BN addition, where the addition of 6% BN increased the passivation of the composite by increasing the values of |Z| and the maximum values of Φ. The presence of 12% BN provides the best passivation against corrosion. It has been reported [30,40,41,42] that metals and alloys with high |Z| values exhibit high corrosion resistance. The same principle is applied to metals and alloys that reveal higher maximum values of Φ. The EIS measurements agreed with the CPP and CCT results that the addition of WC and BN increased the passivation of the AA5083-matrix composite, and the presence of BN provided better protection than WC.

## 4. Conclusions

The corrosion of AA5083-WC and AA5083-BN composites in sodium chloride solution was investigated. The techniques used to investigate the influence of WC and BN additions confirmed the occurrence of general and pitting corrosion in the AA5083-WC/BN composite when exposed to the chloride solution. The presence of 6% WC in the composite decreased the corrosion rate, which led to an increase in its corrosion resistance. This was indicated by decreasing the attained current and changing the pitting potential to a lesser negative value. The addition of 12% WC to the AA5083-matrix remarkably increased its corrosion resistance towards both uniform and pitting attacks. Furthermore, the addition of BN to the AA5083 matrix increases the corrosion resistance by more than 12% WC. The AA5083-12%BN composite exhibited the lowest current and rate of corrosion and revealed the highest resistance to corrosion. All electrochemical measurements were consistent with each other and confirmed that the addition of WC or BN at the examined contents revealed a beneficial influence for protecting the AA5083-matrix composite against corrosion. According to the results of the present study, AA5083-WC and AA5083-BN composites are promising candidates for tribological applications because of the high corrosion resistance of the developed composites, specifically in the case of BN addition.

## Figures and Tables

**Table 1:** The chemical composition of the AA5083 alloy.

Element	Al	Mg	Mn	Cr	Si	Fe	Cu	Zn	Ti	O
Standard (wt.%)	Bal.	4.0–4.9	0.4–1.0	0.05–0.25	≤0.4	≤0.4	≤0.4	≤0.4	≤0.4	≤0.4
Actual (wt.%)	Bal.	4.71	0.7	0.19	0.074	0.16	0.044	0.056	≤0.01	0.19

**Table 2:** The designed composition of the investigated composites in weight percentage.

Composite	Composition		
	AA5083	WC	BN
AA5083 alloy matrix	100	0	0
AA5083 + 6% WC	94	6	0
AA5083 + 12% WC	88	12	0
AA5083 + 6% BN	94	0	6
AA5083 + 12% BN	88	0	12

**Table 3:** Corrosion parameters attained for the composites from the polarization measurements.

Composite	β_c_/ mV·dec^−1^	E_Corr_/ mV	β_a_/ mV·dec^−1^	j_Corr_/ µA·cm^−2^	E_Prot_./ mV	E_Pit_./ mV	R_P_/ Ω·cm^2^	R_Corr_/ mmpy
AA5083 alloy matrix	95	−1060	100	8.5	−760	−705	3492	0.2823
AA5083 + 6% WC	90	−1035	80	0.75	−970	−708	24,552	0.0249
AA5083 + 12% WC	78	−1055	130	2.5	−755	−698	8478	0.0830
AA5083 + 6% BN	120	−910	135	1.9	−840	−685	14,538	0.0631
AA5083 + 12% BN	80	−1095	120	2.3	−800	−695	9078	0.0764

**Table 4:** Impedance data obtained for the tested AA5083 composites in the 3.5% NaCl solution.

Composite	R_S_/Ωcm^2^	Q		R_P1_/ Ω cm^2^	C_dl_/ F cm^−2^	R_P2_/ Ω cm^2^
		Y_Q_/Fcm^−2^	n			
AA5083 alloy matrix	7.309	0.000233	0.68	12.83	0.0000240	7895
AA5083 + 6% WC	22.53	0.000061	0.73	30.33	0.0000020	11,580
AA5083 + 12% WC	16.82	0.000121	0.80	25.86	0.0000035	8980
AA5083 + 6% BN	13.28	0.000110	0.80	33.47	0.0000027	13,240
AA5083+ 12% BN	9.255	0.000149	0.80	26.15	0.0000034	9322

## Data Availability

The experimental datasets obtained from this research work and then the analyzed results during the current study are available from the corresponding author upon reasonable request.
